# Posttraumatic Stress Disorder and Handgrip Strength Among World Trade Center Firefighters and Emergency Medical Responders

**DOI:** 10.3390/ijerph23040413

**Published:** 2026-03-25

**Authors:** Laura Sampson, Yuxiao Song, Frank D. Mann, Rachel Zeig-Owens, Charles B. Hall, Alexandra K. Mueller, Jaeun Choi, Alicia M. Fels, Matthew D. Fajfer, Onix A. Melendez, Christina M. Hennington, Candace W. Arneaud, David J. Prezant, Benjamin J. Luft, Sean A. P. Clouston

**Affiliations:** 1 Program in Public Health Program, Stony Brook University, Stony Brook, NY 11794, USA; yuxiao.song@stonybrook.edu (Y.S.); sean.clouston@stonybrookmedicine.edu (S.A.P.C.); 2 Department of Family, Population, and Preventative Medicine, Renaissance School of Medicine, Stony Brook University, Stony Brook, NY 11794, USA; 3 Department of Medicine, Renaissance School of Medicine, Stony Brook University, Stony Brook, NY 11794, USA; frank.mann@stonybrookmedicine.edu (F.D.M.); benjamin.luft@stonybrookmedicine.edu (B.J.L.); 4 Department of Epidemiology and Population Health, Albert Einstein College of Medicine, Bronx, NY 10461, USA; rachel.zeig-owens@fdny.nyc.gov (R.Z.-O.); charles.hall@einsteinmed.edu (C.B.H.); jaeun.choi@einsteinmed.edu (J.C.); david.prezant@fdny.nyc.gov (D.J.P.); 5 Department of Medicine, Montefiore Medical Center, Bronx, NY 10467, USA; alexandra.mueller@fdny.nyc.gov; 6 Bureau of Health Services, Fire Department of the City of New York, Brooklyn, NY 11201, USA; 7 Saul R. Korey Department of Neurology, Albert Einstein College of Medicine, Bronx, NY 10461, USA; 8 World Trade Center Health Program, Renaissance School of Medicine, Stony Brook University, Stony Brook, NY 11794, USA; alicia.fels@stonybrookmedicine.edu (A.M.F.); onix.melendez@stonybrookmedicine.edu (O.A.M.); christina.hennington@stonybrookmedicine.edu (C.M.H.); candace.arneaud@stonybrookmedicine.edu (C.W.A.)

**Keywords:** posttraumatic stress disorder, handgrip strength, handgrip asymmetry, firefighters and emergency medical responders, PTSD symptom severity

## Abstract

**Highlights:**

**Public health relevance—How does this work relate to a public health issue?**

- PTSD is a long-term and highly prevalent condition among World Trade Center-exposed firefighters and emergency medical responders, a population now entering older age with elevated risk of functional decline.
- Handgrip strength and handgrip asymmetry provide objective indicators of physical function that are linked to disability, cognitive decline, and mortality, making them highly relevant for public health surveillance.

**Public health significance—Why is this work of significance to public health?**

- More than 20 years after 9/11, higher PTSD symptom severity was associated with weaker handgrip strength among FDNY responders, demonstrating a persistent link between psychological trauma and physical function.
- Re-experiencing and avoidance symptoms showed the strongest associations with lower grip strength, indicating that specific PTSD symptom profiles are differentially related to long-term occupational health impairment.

**Public health implications—What are the key implications or messages for practitioners, policy makers and/or researchers in public health?**

- Disaster responder health programs may benefit from integrating routine grip-strength testing with PTSD monitoring to identify individuals at elevated risk for functional decline and disability.
- Occupational and disaster health research should prioritize longitudinal, symptom-specific models to determine whether targeted PTSD interventions can preserve physical function and extend work ability in aging responder populations.

**Abstract:**

Posttraumatic stress disorder (PTSD) has been linked to impaired physical function, which in turn predicts falls, morbidity, and mortality. However, few studies have used objective measures such as handgrip strength to assess physical function. In this cross-sectional study, we investigated associations of average PTSD symptom severity and symptom domain severity with measures of maximum handgrip strength and handgrip asymmetry from 11/2021–12/2023, among 381 male firefighters and emergency medical responders who responded to the World Trade Center disaster, using covariate-adjusted linear regression models. PTSD was diagnosed using the Structured Clinical Interview for the DSM-5 in 17% of responders. Greater overall PTSD average symptom severity was associated with weaker maximum handgrip strength (β = −4.43 lbs; 95%; CI: −8.77, −0.09; *p* = 0.045). Higher re-experiencing symptom severity was associated with weaker maximum handgrip strength (β = −4.17 lbs; 95% CI: −8.13, −0.22; *p* = 0.039). Avoidance symptoms were associated with weaker handgrip strength in adjusted models (β = −4.14 lbs; 95% CI: −7.56, −0.73; *p* = 0.018), and associated with a larger negative difference assessing for hand asymmetry (β = −2.20 lbs; 95% CI: −4.18, −0.22; *p* = 0.029). Findings suggest that PTSD may contribute to long-term physical decline, even in populations with high baseline fitness.

## 1. Introduction

On 11 September 2001 (9/11), the collapse of the Twin Towers at the World Trade Center from a coordinated terrorist attack caused 2997 deaths. First responders to the attacks including firefighters, police officers, and emergency medical services (EMS) personnel aided in the rescue and recovery mission in the days and months after 9/11 and were exposed to traumatic sights, smells, and sounds in addition to hazardous environmental conditions on site [1,2]. Since this event, there has been an expressed interest in understanding the long-term consequences of these exposures on mental and physical health.

Physical function declines with age and is a key marker for overall function and independence in the community [3,4,5,6]. Handgrip strength is a simple yet powerful measure of upper-extremity muscle strength and muscle function and one component of broader physical function assessments; it may also act as a surrogate marker for broader health conditions including cardiovascular disease and mortality [5,7,8,9]. Moreover, weak handgrip strength may be an early indicator of the presence of aging-related disability and may also be a strong predictor of future cognitive impairment [4,8,10]. Similarly, handgrip asymmetry is defined as a larger than expected difference in strength between right and left, or dominant and non-dominant, hands. Prior research suggests that after accounting for maximal handgrip strength, handgrip asymmetry interpedently predicts elevated risk of disability [11], incident medical morbidity [12], and mortality [13] in older adults. One possible explanation is that handgrip dynamometry reflects not only maximal voluntary force generation but also coordinated neuromuscular recruitment and broader brain–muscle communication. In this context, maximum grip strength has been described as a marker of neurological function and brain health, and handgrip asymmetry has also been proposed as a biomarker of neural and neuromuscular integrity. Thus, subclinical neurodegenerative changes, including Lewy body pathology, may plausibly contribute to altered handgrip strength and asymmetry by disrupting the neural systems required for efficient force generation and motor coordination [14,15].

One potential contributor to lower physical function in populations exposed to trauma may be chronic posttraumatic stress disorder (PTSD), a debilitating condition marked by heterogeneous symptoms such as intrusive re-experiencing, emotional, and behavioral responses. For example, among adult veterans, higher PTSD symptom severity was associated with worse physical function across follow-up [16]. However, that study relied on a self-reported measure of physical function (a subscale of the 36-item Short Form Health Survey), which may be subject to biased over-reporting of impairment among individuals with PTSD [17,18]. Emerging evidence using objective measures further suggests that PTSD may also be associated with lower handgrip strength. In one study of children exposed to earthquakes, researchers reported an association between PTSD and lower handgrip strength [19]. Similarly, in a study of non-firefighter responders to the terrorist attacks at the World Trade Center, researchers identified lower than expected handgrip strength for age, concurrent with slower walk speed, slower chair rise speed, and worse cognitive performance; these deficits were especially pronounced among those with more severe re-experiencing symptoms (i.e., nightmares and flashbacks) [20,21]. However, the causal pathways linking PTSD to reduced handgrip performance remain unclear and may involve downstream behavioral and physiological processes rather than a direct effect alone.

Previous studies have shown that PTSD is associated with maximal handgrip strength when adjusting for other symptom types as well as the presence of depression [20]. However, relatively little is known about the potential mechanisms underlying reduced muscle function and related physical functional impairment in trauma-exposed populations with PTSD. Moreover, given that prior studies have noted associations between intense nightmares and neurological conditions such as Lewy body dementia and Parkinson’s disease [22,23,24], a potential association between handgrip asymmetry and PTSD could help explain observed correlations between PTSD symptoms, including nightmares, and impaired physical functioning overall. Additionally, relatively little is known about the relationship between PTSD and handgrip strength in firefighters, whose baseline physical fitness levels and occupational experiences differ from those of veterans and other first responders. Given prior work supporting handgrip strength as an objective indicator of muscle function and decline within broader physical function [3,5,10], in the present study, we examined the relationships of PTSD diagnosis, overall PTSD symptom severity, and PTSD symptom domain severity with objective measures of maximum handgrip strength and handgrip asymmetry in a sample of male Fire Department of the City of New York (FDNY) responders to the 9/11 terrorist attacks. We hypothesized that PTSD would be associated with lower maximum handgrip strength and greater handgrip asymmetry. Moreover, given hypothesized mechanisms and prior work among general responders, we expected that re-experiencing symptoms would be most strongly associated with these outcomes.

## 2. Materials and Methods

### 2.1. Setting

Recruitment for this cross-sectional study was conducted in collaboration with the FDNY Health Monitoring Program and has been previously described [25]. Briefly, eligible members were contacted based on prior WTC site deployment records and invited to participate during their periodic health surveillance appointments. Additionally, participants could be referred by other responders or by clinical staff when necessary. Included participants completed a standardized PTSD assessment and underwent handgrip strength testing.

### 2.2. Inclusion Criteria

Participants must have been an FDNY employee with documented exposures to the WTC disaster sites between 9/11/2001–7/2002. Surviving responders were eligible for study enrollment if they participated in the FDNY WTC Health Program, resided near Long Island, New York, had valid contact information on file, and were willing to travel to clinic sites to participate in research. Data for this study were collected over a two-year period, spanning 11/2021–12/2023.

Responders were excluded if they had missing data on the primary exposure (PTSD assessment) or primary outcomes (handgrip strength indicators), or if they had a current or past diagnosis of neurological conditions (e.g., brain cancer, stroke, or traumatic brain injury) that could affect cognitive or physical performance, consistent with prior FDNY cohort studies. The variable for occupation was based on role at the time of 9/11, recognizing that some EMS personnel later transitioned to firefighter roles. To support a reliable estimation, female responders (*n* = 6) were also excluded due to the small sample size. The final analytic sample included 381 male responders with complete data. A non-parametric test for missing-data mechanisms was not statistically significant (*p* = 0.629), supporting the assumption that data were missing completely at random (MCAR) [26].

### 2.3. Ethics

The study protocol was approved by the Stony Brook University Institutional Review Board CORIHS IRB #2021-00295, effective from 27 July 2021. Participants provided written informed consent prior to data collection.

### 2.4. Measures

#### 2.4.1. Assessment of Posttraumatic Stress Disorder (PTSD) Symptoms

PTSD was assessed using the Structured Clinical Interview for the Diagnostic and Statistical Manual of Mental Disorders, Fifth Edition (SCID) [27]. Diagnosis of current PTSD was recorded by trained staff and reviewed by the lead study administrator for quality control purposes. Where feasible, SCIDs were recorded for training and quality control purposes. During administration, PTSD symptoms were collected by interviewers within five main symptom domains: re-experiencing, avoidance, numbing, hyperarousal and negative thoughts. For each symptom, symptom severity was scored: 1 = Symptom absent, 2 = Present but not clinically significant, and 3 = Clinically significant. For each domain, a domain score was computed as the mean of its constituent items (range, 1–3), with higher values indicating greater severity. An overall average PTSD symptom severity score (Cronbach’s α = 0.780; 95% CI = 0.74, 0.81) was computed by averaging across all individual symptom items spanning the five domains, consistent with prior WTC responder studies using SCID-based PTSD assessments [20,25]. This approach captures the overall severity of PTSD symptomatology while maintaining comparability with previous analyses.

#### 2.4.2. Handgrip Strength

We measured handgrip strength using a Vernier dynamometer, following standardized protocols in previous studies [20,28]. Participants were seated with feet flat, the test arm at the side, the elbow at 90°, and the forearm neutral. Each hand performed two maximal voluntary contractions with brief rest between trials; testing was deferred for participants reporting upper-extremity pain, injury, or functional limitation. For each hand, the higher of the two trials (in lbs) was retained for analysis. Before testing, participants self-reported hand dominance as left, right, or ambidextrous. From the best-trial values, we defined the maximum handgrip strength as the higher value of the two hands’ best trials (lbs), providing a single summary of overall grip capacity.

#### 2.4.3. Handgrip Asymmetry

Handgrip asymmetry was derived from the best trial from each hand. The primary asymmetry outcome was the dominant-to-non-dominant handgrip asymmetry (lbs), as the difference between the best dominant-hand value and the best non-dominant-hand value. Negative values indicated that the non-dominant hand was stronger than the dominant hand. Among ambidextrous participants, asymmetry was quantified using the absolute grip strength difference between hands. As a supplementary asymmetry outcome, we examined the absolute left–right handgrip asymmetry (lbs), irrespective of hand dominance. Results from this supplementary metric were directionally consistent with those from the primary metric but showed wider confidence intervals and slightly larger standard errors; therefore, it is presented in the Supplementary Materials.

#### 2.4.4. Covariates

We reported both sociodemographic and health-related variables descriptively and accounted for sociodemographic covariates in regression models to control for potential confounding in the associations between PTSD and handgrip strength outcomes. These included participants’ age (modeled continuously in years after testing for nonlinearity), self-reported race/ethnicity (categorized as White vs. Non-White due to small numbers of individual Non-White races), education level (high school, some college/technical school, or university degree and above), and occupational role at the time of 9/11 (firefighter, EMS). Hand dominance (right-handed, left-handed, or ambidextrous), self-reported major depressive disorder (assessed using the Patient Health Questionnaire, PHQ-9), physician-diagnosed asthma, cardiovascular disease, hypertension, and diabetes were abstracted from the clinical record and summarized descriptively in Table 1; these comorbidities were not included in regression models given potential mediation and uncertain temporality.

#### 2.4.5. Statistical Analysis

All analyses were performed using RStudio [V.4.3.1]. Descriptive statistics summarized sample characteristics, overall and stratified by PTSD status. Characteristics of categorical variables were reported as frequencies and percentages, while continuous variables were summarized using means and standard deviations or medians and interquartile ranges, depending on the underlying distribution. Figures including violin plots were generated using the ggplot2 package, and tables were constructed using gtsummary package in RStudio. We estimated associations between PTSD symptom severity and each handgrip strength outcome using linear regression models specified in two stages: (1) unadjusted models and (2) multivariable-adjusted models including age, race/ethnicity, education, and occupation. Maximum handgrip strength and handgrip asymmetry were modeled as continuous outcomes because no well-established clinically meaningful cut-points exist for these measures in this population, and categorization could reduce statistical power and result in loss of information. For key multivariable-adjusted models, we also generated marginal prediction plots with 95% confidence intervals to visualize predicted values of each handgrip outcome across observed levels of PTSD symptom severity. To avoid potential mediation, major depressive disorder and hand dominance were not included as covariates in the regression models. Regression estimates are presented as β coefficients with corresponding 95% confidence intervals (CIs) and *p*-values. Specific PTSD symptom domain severity scores were entered individually to assess associations with each outcome. As a sensitivity analysis, we repeated the fully adjusted models after stratifying the sample by age (<65 vs. ≥65 years) included in the Supplementary Materials. The missing-data mechanism was evaluated using a distribution-free procedure appropriate for both normal and non-normal variables [26].

## 3. Results

**Figure 1:**
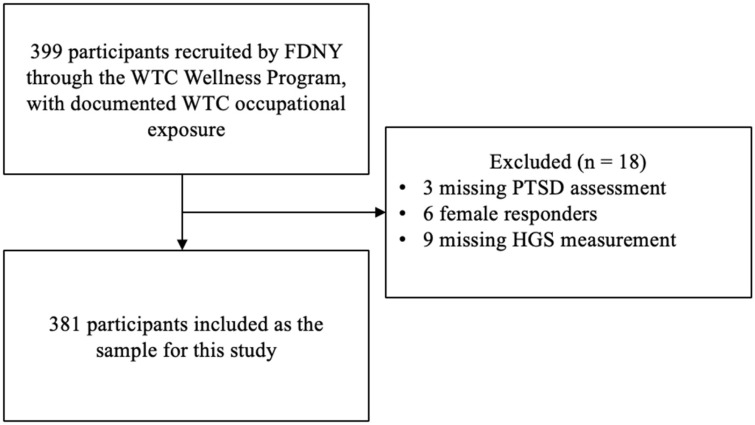
Flowchart depicting participant selection process for this cross-sectional study examining associations between post-traumatic stress disorder (PTSD) symptoms and handgrip strength (HGS) outcomes among Fire Department of the City of New York (FDNY) responders to the 9/11 World Trade Center (WTC) attacks.

Among 399 FDNY responders in this WTC Health Program study, 18 were excluded (nine without handgrip strength measurements, six female responders, and three without PTSD assessment), yielding an analytic cohort of 381 men with complete PTSD measures, hand dominance, and handgrip strength (Figure 1).

The characteristics of the cohort are summarized in Table 1. The analytic sample included 381 responders, among whom 66 (17%) were diagnosed with PTSD. The median age was 60 years (IQR, 55–65). Most responders were White (94%), right-hand dominant (85%), and firefighters (94%); 38% held a university degree or higher. Clinical-diagnosed asthma (41%) and hypertension (26%) were the most common chronic conditions. Compared with responders without PTSD, those with PTSD were less likely to have a university degree or higher (27% vs. 40%) and were more likely to have asthma (55% vs. 38%), hypertension (30% vs. 25%), and major depressive disorder (23% vs. 2.2%). Maximum handgrip strength was lower among responders with PTSD than among those without PTSD (60.3 vs. 63.1 lbs). The dominant-to-non-dominant grip strength difference averaged 3.3 lbs (SD, 8.8) overall and differed little by PTSD status (3.2 lbs in responders with PTSD vs. 3.3 lbs in responders without PTSD). In contrast, mean absolute left-to-right handgrip asymmetry was greater among responders with PTSD than among those without PTSD (8.4 vs. 7.1 lbs).

Additional subgroup means for handgrip strength outcomes are reported in Supplemental Table S1. Maximum handgrip strength was highest among responders with a university degree or higher (64.9 lbs) and lowest among those with diabetes (54.4 lbs). Responders with cardiovascular disease (58.0 lbs), asthma (60.1 lbs), or hypertension (60.1 lbs) also had lower maximum handgrip strength. By occupation, firefighters averaged 62.8 lbs, compared with 60.0 lbs among EMS personnel. By hand dominance, right-hand dominant responders averaged 63.2 lbs, left-hand dominant responders 60.5 lbs, and ambidextrous responders 57.3 lbs.

The dominant-to-non-dominant grip strength difference was greatest among responders with diabetes (8.9 lbs) and among ambidextrous responders (7.1 lbs), and smallest among those with cardiovascular disease (1.7 lbs) and those with a high school education (−0.6 lbs). Absolute left-to-right handgrip asymmetry, examined as a supplementary metric, was greatest among responders with comorbid PTSD and major depressive disorder (9.1 lbs) and among those with diabetes (8.9 lbs), and was smallest among responders with major depressive disorder only (3.5 lbs).

### 3.1. Associations Between PTSD and Handgrip Strength

**Figure 2:**
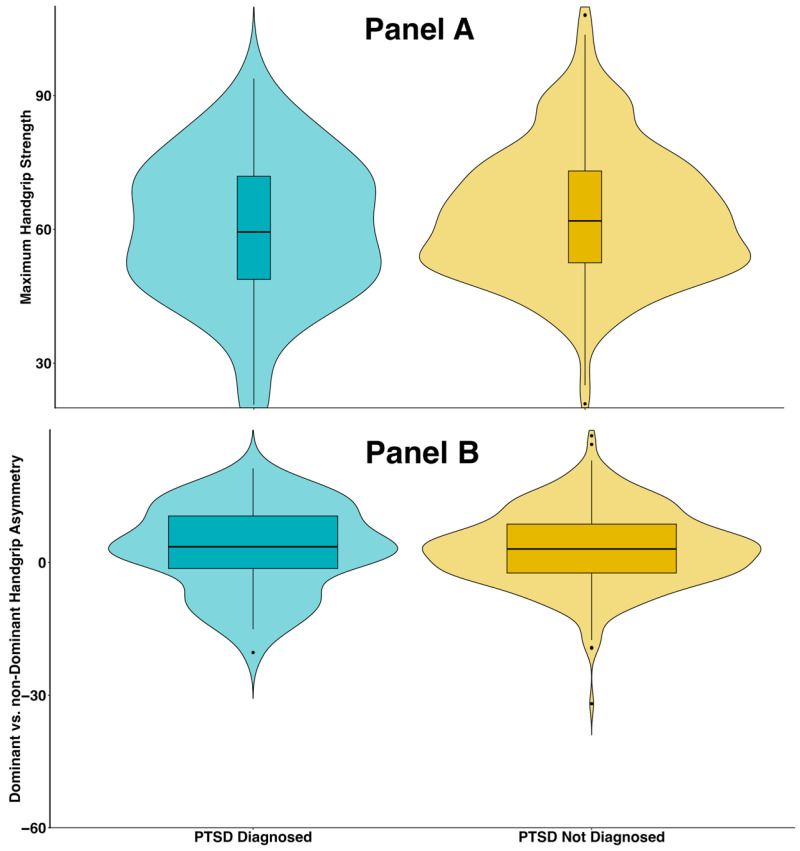
Violin plots illustrating the distribution of handgrip strength outcomes stratified by post-traumatic stress disorder (PTSD) diagnosis status, among 381 male responders from the Fire Department of the City of New York (FDNY). Panel (**A**): Maximum handgrip strength by PTSD diagnosis. Panel (**B**): Dominant vs. non-dominant handgrip asymmetry by PTSD diagnosis.

Responders diagnosed with PTSD had lower maximum handgrip strength than those without PTSD (60.3 lbs vs. 63.1 lbs; Figure 2A). The violin plots display the full distribution of values, with the horizontal line indicating the median. The dominant-to-non-dominant grip strength difference was similar across PTSD groups, with slightly lower values among responders with PTSD than among those without PTSD (3.2 lbs vs. 3.3 lbs; Figure 2B). Sensitivity analyses using absolute left-to-right handgrip asymmetry as a supplementary metric (Supplemental Figure S1) yielded similar findings.

**Figure 3:**
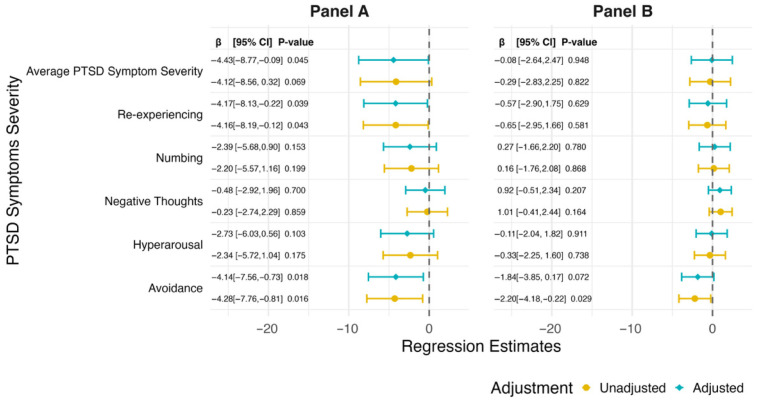
Associations of posttraumatic stress disorder (PTSD) symptom severity with handgrip strength outcomes among 381 male responders from the Fire Department of the City of New York (FDNY), shown by model adjustment levels. Estimates are shown for unadjusted (bottom circle) and multivariable adjusted (top diamond) models. Panel (**A**): Maximum handgrip strength by PTSD diagnosis. Panel (**B**): Dominant vs. non-dominant handgrip asymmetry by PTSD diagnosis.

We then examined associations between PTSD symptom severity variables and maximum handgrip strength (Figure 3A). In the multivariable-adjusted models, greater avoidance symptom severity was significantly associated with lower maximum handgrip strength (β = −4.14; 95% CI, −7.56 to −0.73; *p* = 0.018). Similar inverse associations were observed for re-experiencing symptoms (β = −4.17; 95% CI, −8.13 to −0.22; *p* = 0.039) and for average PTSD symptom severity (β = −4.43; 95% CI, −8.77 to −0.09; *p* = 0.045). Maximum handgrip strength was not significantly associated with numbing symptoms (β = −2.39; 95% CI, −5.68 to 0.90; *p* = 0.153), hyperarousal symptoms (β = −2.73; 95% CI, −6.03 to 0.56; *p* = 0.103), or negative thoughts symptoms (β = −0.48; 95% CI, −2.92 to 1.96; *p* = 0.700).

We also examined the dominant-to-nondominant grip strength difference (Figure 3B). In the unadjusted model, greater avoidance symptom severity was associated with a more negative dominant-to-nondominant grip strength difference (β = −2.20; 95% CI, −4.18 to −0.22; *p* = 0.029). This association was attenuated and no longer statistically significant after multivariable adjustment (β = −1.84; 95% CI, −3.85 to 0.17; *p* = 0.072), although the direction of the association remained similar. No other PTSD symptom domains, nor average PTSD symptom severity, were significantly associated with the dominant to non-dominant grip strength difference in either the unadjusted or multivariable-adjusted models.

**Figure 4:**
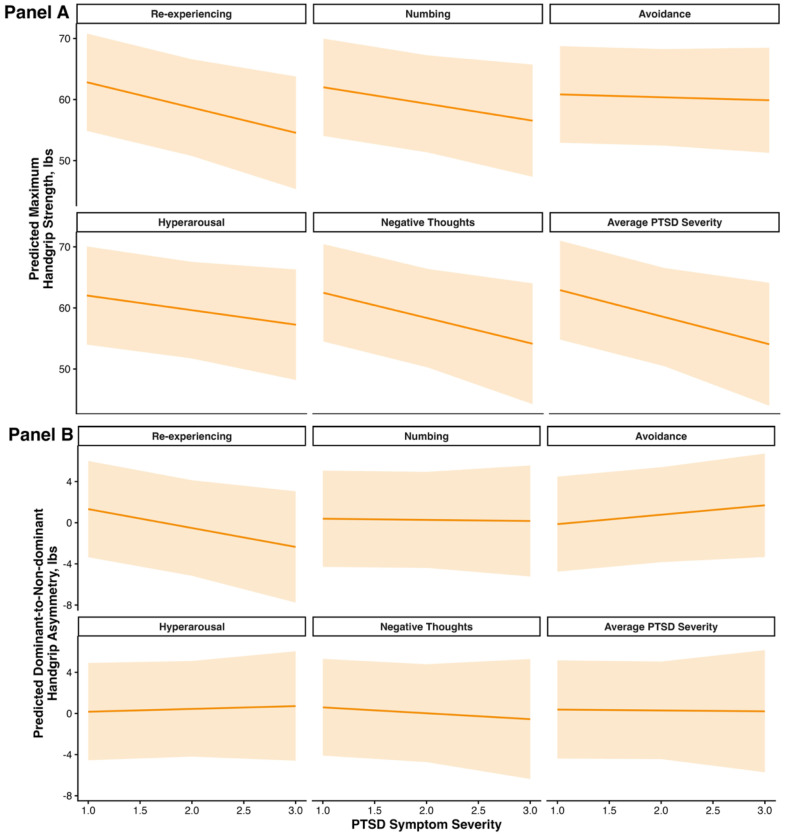
Multivariable-adjusted marginal predictions of maximum handgrip strength and dominant-to-nondominant grip strength difference across levels of PTSD symptom severity among 381 male Fire Department of the City of New York (FDNY) responders. Facets represent individual PTSD symptom domains and average PTSD symptom severity. Shaded areas indicate 95% confidence intervals. Panel (**A**): Maximum handgrip strength by PTSD Symptom Severity. Panel (**B**): Dominant vs. non-dominant handgrip asymmetry by PTSD Symptom Severity.

We then further assessed these associations using multivariable-adjusted marginal prediction plots (Figure 4). Across the observed symptom range, predicted maximum handgrip strength decreased from 62.8 to 54.6 lbs for re-experiencing symptoms, from 62.0 to 56.5 lbs for numbing symptoms, from 60.8 to 59.9 lbs for avoidance symptoms, from 62.0 to 57.2 lbs for hyperarousal symptoms, from 62.5 to 54.1 lbs for negative thoughts symptoms, and from 62.9 to 54.1 lbs for average PTSD symptom severity. By contrast, dominant vs. non-dominant handgrip asymmetry changed more modestly across symptom severity levels, ranging from a 3.68-lb decrease for re-experiencing symptoms to a 1.83-lb increase for avoidance symptoms, with smaller changes observed for the remaining domains and broad overlap in the confidence intervals.

### 3.2. Sensitivity Analyses

Supplemental Figure S2 presents the associations between PTSD symptom severity and absolute left-to-right handgrip asymmetry. Across both unadjusted and multivariable-adjusted models, no PTSD symptom domain or average PTSD symptom severity was significantly associated with this supplementary asymmetry outcome. In the multivariable-adjusted models, estimates were near null for average PTSD symptom severity (β = 0.24; 95% CI, −0.72 to 1.20; *p* = 0.622), re-experiencing symptoms (β = 0.40; 95% CI, −0.90 to 1.69; *p* = 0.546), avoidance symptoms (β = 0.84; 95% CI, −0.87 to 2.55; *p* = 0.336), and hyperarousal symptoms (β = −0.26; 95% CI, −1.61 to 1.09; *p* = 0.705). Numbing symptoms (β = 1.01; 95% CI, −0.55 to 2.57; *p* = 0.203) and negative thoughts symptoms (β = 1.11; 95% CI, −0.19 to 2.40; *p* = 0.093) also were not significantly associated with absolute left-to-right handgrip asymmetry.

Supplemental Figures S3 and S4 present multivariable-adjusted models stratified by age group. For maximum handgrip strength, inverse associations were generally observed in both strata, although all confidence intervals crossed the null. The largest negative estimates were observed for re-experiencing symptoms among responders aged ≥65 years (β = −3.95; 95% CI, −9.77 to 1.87; *p* = 0.181). Among responders aged <65 years, the largest negative estimates were observed for numbing symptoms (β = −4.42; 95% CI, −9.41 to 0.56; *p* = 0.082) and hyperarousal symptoms (β = −3.88; 95% CI, −8.08 to 0.31; *p* = 0.069). For dominant-to-non-dominant handgrip asymmetry and absolute left-to-right handgrip asymmetry, age-stratified estimates varied in direction but remained imprecise, with all confidence intervals crossing the null in both age groups.

## 4. Discussion

There is an increasing concern that individuals with chronic PTSD after a traumatic event may be experiencing more rapid aging and that this aging may be evidenced by the presence of worsened physical functioning. In this cross-sectional study of 381 male FDNY personnel who responded to the 9/11 WTC terrorist attacks, we found that—more than 20 years after the attacks—PTSD overall and re-experiencing symptoms specifically were associated with weaker handgrip strength. We also found that more severe avoidance symptoms were associated with weaker maximal handgrip strength and with more handgrip asymmetry. Overall, our findings replicate results reported in other cohorts and also extend them to include variation in handgrip asymmetry.

This is the first study to assess the relationships between PTSD and handgrip strength outcomes among FDNY WTC responders, and one of the only studies in any population to assess PTSD and handgrip asymmetry. Nevertheless, our findings are largely consistent with prior results in WTC “general responders” who comprised primarily law enforcement personnel [20]. The average maximum handgrip strength was slightly higher in our FDNY sample (62.6 lbs compared to 57.4 lbs in the general responder study), despite a slightly older sample (mean age approximately 60 years vs. 53 years), which could be indicative of baseline physical fitness differences related to occupation. However, both groups demonstrated lower handgrip strength than might be expected for their age, sex, and occupations. Normative data suggest that handgrip strength for this age group of civilian men is typically over 100 lbs [29,30,31]. These differences could be due to PTSD-related accelerated aging via chronic inflammation, hypothalamic–pituitary–adrenal (HPA) axis dysregulation, and possible neuroinflammation among responders [16,32,33,34,35,36,37,38,39,40,41,42], or possibly related to environmental exposures experienced on site during rescue and recovery, including inhalation of toxic pollutants [43]. However, since this study used a different dynamometer than is used in normative populations, any differences should be interpreted with caution.

We found an association between PTSD symptom severity with weaker maximum handgrip strength, consistent with prior studies [20,28]. Individual domain symptom results differed slightly, with our study showing re-experiencing and avoidance symptom severity associated with weaker handgrip strength, whereas prior research found that re-experiencing symptoms were the only symptom domain to be statistically significantly associated with handgrip strength when mutually adjusting for the other domains [20]. Both re-experiencing and hyperarousal symptoms are more physical symptoms that point to a potential biological mechanism like HPA axis dysregulation, which can lead to elevated levels of cortisol and systemic inflammation, both of which may contribute to accelerated biological aging and resulting sarcopenia [32,33,44]. However, avoidance symptoms are a more social or behavioral manifestation of PTSD, and may suggest that physical disengagement or behavioral withdrawal could also play a role in driving physical functional decline and worse handgrip performance. Longitudinal research has noted that the re-experiencing symptoms often dissipate over time after the trauma while avoidance symptoms become more important over time [45]. Thus, an alternative interpretation of this work may be that avoidance symptoms may serve as a marker to capture a later or more persistent manifestation of PTSD severity. Future work should assess these possible mechanisms by collecting longitudinal data including repeated measures of handgrip strength over time and assess variables that may lie on the pathway from PTSD to lower handgrip strength, like loss of muscle mass, physical activity, inflammation markers, neuroinflammation measures, and even sleep quality over time, to disentansgle possible mediating pathways. Additionally, future work might examine patterns across repeated handgrip efforts—for example, whether the second attempt is systematically higher among individuals without PTSD—as emerging evidence from other clinical populations suggests that trial-to-trial changes in handgrip strength may capture fatigue-related patterns beyond what a single maximum value reflects, with potential relevance for trauma-exposed populations [46,47]. Another promising direction for future research is to enroll a control group of external firefighters to more accurately determine whether the association we observed is specific to firefighters with World Trade Center-related exposure. Firefighters typically exhibit greater psychological and physiological resilience than the general population, resulting in lower reported PTSD rates despite repeated exposure to traumatic events [48,49]. Accounting for this baseline occupational differences would clarify whether the PTSD-grip strength associations are specific to World Trade Center-related exposed firefighters.

### Strengths and Limitations

The primary strength of this study was our use of objective handgrip strength as a measure of physical function and aging, which produces less bias than subjective recall of physical function [17,18]. We also used a gold standard diagnosis measure of PTSD [27], in addition to individual symptom domain severity, which provided insight into specific facets of PTSD that may drive functional impairment. The primary limitation of our study is its cross-sectional design; we cannot determine with certainty whether PTSD caused lower physical function or whether alternatively, poor physical health may have exacerbated PTSD symptoms. However, our unique population and study design in some ways alleviates this concern, since we know that responders had to be physically strong and fit before the 9/11 attacks to be serving as firefighters, making it unlikely that physical functional decline preceded PTSD from 9/11. As a second limitation, although our sample size was sufficient for detecting small-to-moderate associations, some estimates were imprecise with wide confidence intervals, which may have resulted in lack of statistical significance for some relationships. Third, given the very small number of women in our study, we excluded them from analyses. However, this issue reflects the even smaller proportion of women in the overall FDNY, so results remain generalizable to the FDNY population but may not generalize to other populations. Fourth, though all FDNY WTC responders are eligible for free treatment and care for PTSD and related illnesses, we could not account for the variability in treatment accessed or used by these responders. Firefighters have higher physical and psychological resilience than other populations and therefore tend to have lower levels of PTSD despite traumatic lifetime exposures. Future work may also investigate the possible impact of heightened resilience in suppressing the associations identified in this study, or of PTSD treatment trajectories—particularly interventions targeting specific symptom types—on physical function outcomes, which may offer insights for preserving physical health and independence in aging, trauma-exposed populations. Finally, while the presence of handgrip weakness and handgrip asymmetry are putative indicators of cortical Lewy bodies, we did not assess measures of neuropathology in this study.

## 5. Conclusions

In summary, we found that PTSD was associated with lower handgrip strength among male FDNY WTC responders more than two decades after their occupational exposure to 9/11, replicating prior results from WTC general responders. While re-experiencing symptoms were hypothesized to be most predictive of impairment, avoidance symptoms also emerged as a consistent correlate of both weaker handgrip strength and greater handgrip asymmetry in this study. Clinically, these results may imply that individuals with both re-experiencing and avoidance symptoms are at increased risk of downstream outcomes including disability. Our findings and others also underscore the importance of integrating physical performance monitoring into long-term care for aging, trauma-exposed individuals, even in populations with high baseline physical fitness like this one, given that handgrip strength in aging is predictive of future cognitive impairment, physical disability, chronic conditions and mortality.

## Figures and Tables

**Table 1:** Sample characteristics for 381 male Fire Department for the City of New York (FDNY) responders, overall and by post-traumatic stress disorder (PTSD) status.

Variable	Overall N = 381 ^1^	PTSD Diagnosed vs. PTSD Not Diagnosed	
		Yes N = 66 ^1^	No N = 315 ^1^
Maximum Handgrip Strength, lbs †	62.6 (15.4)	60.3 (15.9)	63.1 (15.3)
Handgrip Asymmetry, lbs (Dominant-to-Non-dominant) †	3.3 (8.8)	3.2 (9.7)	3.3 (8.6)
Handgrip Asymmetry, lbs (Absolute Left-to-Right) †	7.3 (5.8)	8.4 (5.7)	7.1 (5.8)
Age, years	60.0 (55.0, 65.0)	59.0 (55.0, 64.0)	60.0 (55.0, 65.0)
Age, category			
<65	281 (74%)	52 (79%)	229 (73%)
≥65	100 (26%)	14 (21%)	86 (27%)
Occupation			
Firefighter	357 (94%)	62 (94%)	295 (94%)
Emergency Medical Services	24 (6.3%)	4 (6.1%)	20 (6.3%)
Race/Ethnicity			
Non-White	23 (6.0%)	5 (7.6%)	18 (5.7%)
White	358 (94%)	61 (92%)	297 (94%)
Education			
High School	42 (11%)	13 (20%)	29 (9.2%)
Some College/Technical School	194 (51%)	35 (53%)	159 (50%)
University degree or Higher	145 (38%)	18 (27%)	127 (40%)
Hand Dominance			
Left	42 (11%)	9 (14%)	33 (10%)
Right	325 (85%)	54 (82%)	271 (86%)
Ambidextrous	14 (3.7%)	3 (4.5%)	11 (3.5%)
Major Depressive Disorder			
Yes	22 (5.8%)	15 (23%)	7 (2.2%)
Asthma			
Yes	156 (41%)	36 (55%)	120 (38%)
Diabetes			
Yes	14 (3.7%)	3 (4.5%)	11 (3.5%)
Cardiovascular Disease			
Yes	32 (8.9%)	3 (4.7%)	29 (9.8%)
Hypertension			
Yes	100 (26%)	20 (30%)	80 (25%)
Average PTSD Symptom Severity	1.4 (0.3)	1.9 (0.3)	1.2 (0.2)
Numbing Symptoms	1.3 (0.5)	2.0 (0.6)	1.2 (0.3)
Avoidance Symptoms	1.4 (0.6)	2.1 (0.7)	1.2 (0.5)
Re-experiencing Symptoms	1.4 (0.4)	1.9 (0.4)	1.3 (0.4)
Hyperarousal Symptoms	1.4 (0.5)	2.0 (0.4)	1.3 (0.3)
Negative thoughts Symptoms	1.3 (0.4)	1.7 (0.5)	1.2 (0.3)
Comorbid PTSD & Major Depressive Disorder			
Neither	308 (81%)	0 (0%)	308 (98%)
PTSD only	51 (13%)	51 (77%)	0 (0%)
MDD only	7 (1.8%)	0 (0%)	7 (2.2%)
Comorbid PTSD & MDD	15 (3.9%)	15 (23%)	0 (0%)

^1^ Median (Interquartile Range) or Frequency (%). † The measurements in handgrip strength, being normally distributed, is presented as the mean (standard deviation).

## Data Availability

Due to ethical, legal, and privacy restrictions related to the World Trade Center (WTC) Fire Department of the City of New York (FDNY) responder cohort, the data are not publicly available.

## Supplementary Materials

The following supporting information can be downloaded at: https://www.mdpi.com/article/10.3390/ijerph23040413/s1. Table S1: Sample summary table for maximum handgrip strength and handgrip asymmetry (lbs) for 381 Fire Department of the City of New York (FDNY) male responders; Figure S1: Violin plot illustrating the distribution of handgrip asymmetry comparing absolute left-to-right handgrip strength, stratified by post-traumatic stress disorder (PTSD) diagnosis status, among 381 male responders from the Fire Department of the City of New York (FDNY); Figure S2: Associations between post-traumatic stress disorder (PTSD) symptom severity and absolute left-to-right handgrip strength outcomes in 381 male responders from the Fire Department of the City of New York (FDNY), shown by model adjustment levels. Estimates are shown for unadjusted (bottom circle), and multivariable-adjusted (top diamond) models. Figure S3: Multivariable-adjusted linear regression estimates showing the associations between PTSD symptom severity and handgrip strength outcomes in 381 male responders from the Fire Department of the City of New York (FDNY), stratified by age group (<65 vs. ≥65). Estimates are shown by age group: <65 (bottom circle) and ≥65 (top triangle). Panel A: Maximum handgrip strength by PTSD diagnosis. Panel B: Dominant vs. non-dominant handgrip asymmetry by PTSD diagnosis. Figure S4: Multivariable-adjusted linear regression estimates showing the associations between PTSD symptom severity and absolute left-to-right handgrip asymmetry in 381 male responders from the Fire Department of the City of New York (FDNY), stratified by age group (<65 vs. ≥65). Estimates are shown by age group: <65 (bottom circle) and ≥65 (top triangle).

## Abbreviations

The following abbreviations are used in this manuscript:

FDNY	Fire Department of the City of New York
PTSD	Posttraumatic stress disorder
SCID	Structured Clinical Interview for the Diagnostic and Statistical Manual of Mental Disorders
WTC	World Trade Center
