# Supplementary material for: Posttraumatic Stress Disorder and Handgrip Strength Among World Trade Center Firefighters and Emergency Medical Responders
Source: Int J Environ Res Public Health. 2026 Mar 25;23(4):413. doi: 10.3390/ijerph23040413 (PMC13116488; doi:10.3390/ijerph23040413)
Supplement: Supplementary file 1 [file ijerph-23-00413-s001.zip › ijerph-4099269-supplementary.pdf]

Supplementary Materials

Table S1. Sample summary table for maximum handgrip strength and handgrip asymmetry (lbs) for 381 Fire Department of the City of New York (FDNY) male responders.

Characteristics	Mean (SD)<br>Maximum Handgrip Strength, lbs	Handgrip Asymmetry, Dominant to Non-Dominant, lbs	Handgrip Asymmetry, Absolute Left-to-Right, lbs
Occupation			
Firefighter	62.8 (15.3)	3.2 (8.8)	7.4 (5.8)
Emergency Medical Services	60.0 (18.1)	3.8 (8.4)	7.0 (5.9)
Race/Ethnicity			
Non-White	62.5 (10.8)	4.1 (8.1)	7.5 (5.0)
White	62.6 (15.7)	3.2 (8.8)	7.3 (5.9)
Education			
High School	57.3 (13.0)	-0.6 (9.6)	7.8 (5.6)
Some College/Technical School	62.1 (15.3)	4.1 (8.1)	7.0 (5.8)
University degree or Higher	64.9 (16.0)	3.4 (9.1)	7.7 (5.9)
Hand dominance			
Left	60.5 (16.2)	0.9 (8.5)	6.9 (5.0)
Right	63.2 (15.3)	3.4 (8.8)	7.4 (5.9)
Ambidextrous	57.3 (15.7)	7.1 (7.2)	7.1 (7.2)
Major Depressive Disorder			
Yes	61.1 (14.2)	2.7 (8.9)	7.3 (5.5)
No	62.7 (15.5)	3.3 (8.8)	7.3 (5.9)
Asthma			
Yes	60.1 (15.4)	2.7 (9.9)	8.0 (6.4)
No	64.4 (15.3)	3.7 (7.9)	6.9 (5.4)
Diabetes (Type I & II)			
Yes	54.4 (16.4)	8.9 (8.4)	8.9 (8.4)
No	63.0 (15.3)	3.1 (8.7)	7.3 (5.7)
Cardiovascular Disease			
Yes	58.0 (13.5)	1.7 (8.0)	6.6 (4.8)
No	63.1 (15.6)	3.4 (8.8)	7.4 (5.9)
Hypertension			
Yes	60.1 (15.3)	3.5 (8.9)	7.3 (6.1)
1No	63.5 (15.4)	3.2 (8.8)	7.4 (5.7)
Comorbid PTSD & Major Depressive Disorder			
Neither	63.2 (15.4)	3.3 (8.7)	7.2 (5.9)
PTSD only	60.0 (16.3)	3.2 (9.5)	8.2 (5.8)
MDD only	60.2 (13.5)	2.1 (3.9)	3.5 (2.6)
Comorbid PTSD & MDD	61.5 (15.0)	2.9 (10.6)	9.1 (5.6)

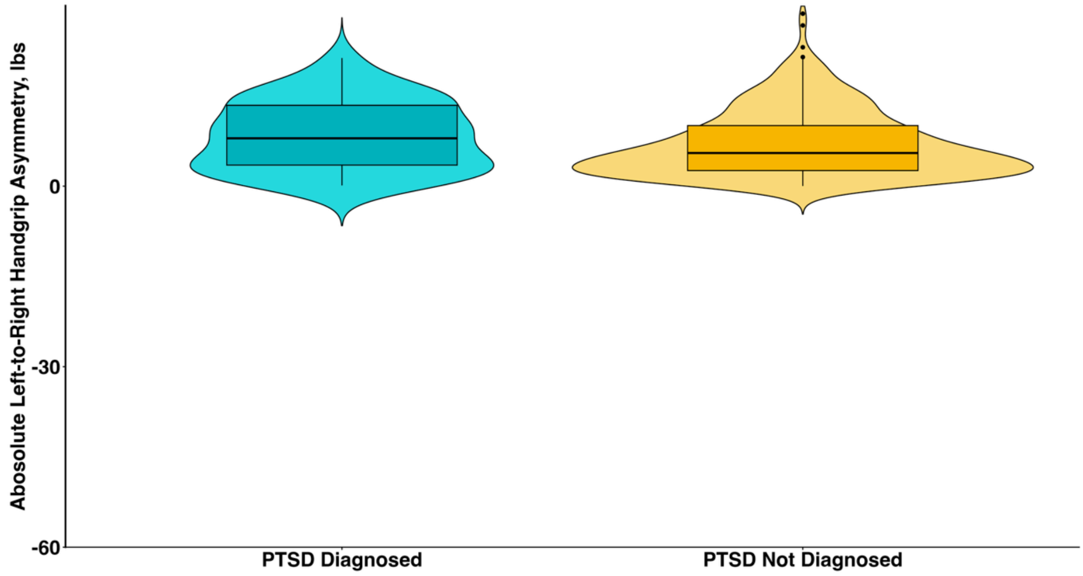

Figure S1. Violin plot illustrating the distribution of handgrip asymmetry comparing absolute left-to-right handgrip strength, stratified by post-traumatic stress disorder (PTSD) diagnosis status, among 381 male responders from the Fire Department of the City of New York (FDNY).

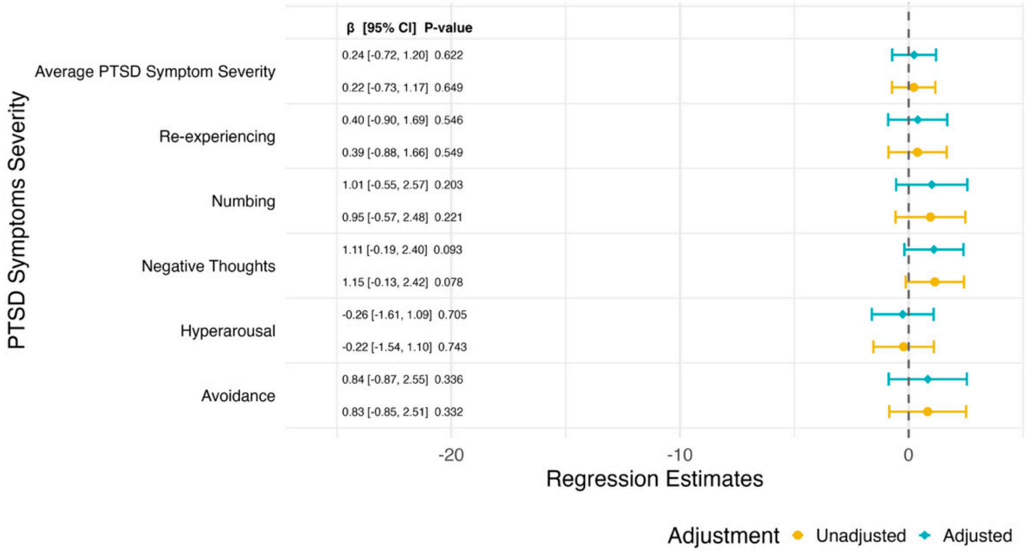

Figure S2. Associations between post-traumatic stress disorder (PTSD) symptom severity and absolute left-to-right handgrip strength outcomes in 381 male responders from the Fire Department of the City of New York (FDNY), shown by model adjustment levels. Estimates are shown for unadjusted (bottom circle), and multivariable-adjusted (top diamond) models.

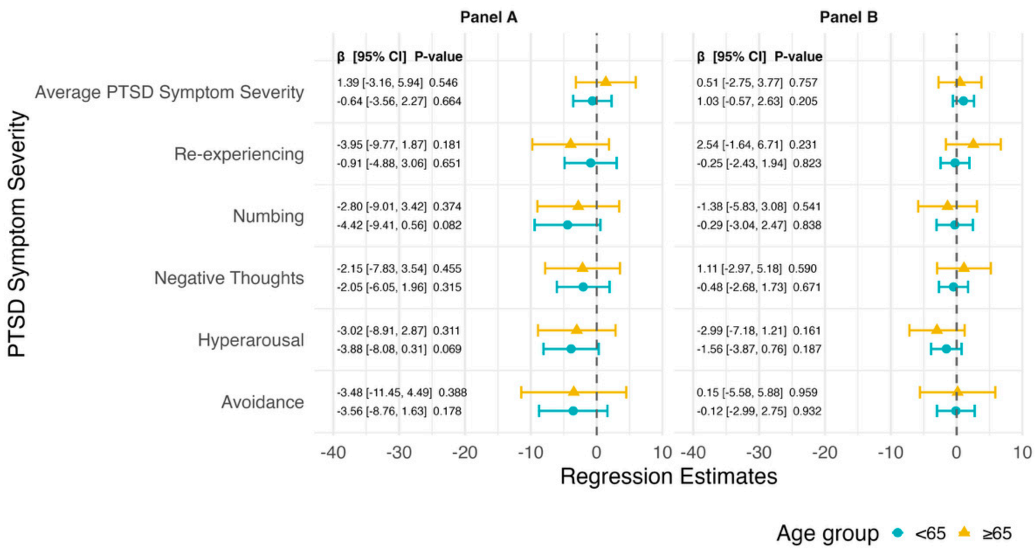

Figure S3. Multivariable-adjusted linear regression estimates showing the associations between PTSD symptom severity and handgrip strength outcomes in 381 male responders from the Fire Department of the City of New York (FDNY), stratified by age group (<65 vs. ≥65). Estimates are shown by age group: <65 (bottom circle) and ≥65 (top triangle). Panel A: Maximum handgrip strength by PTSD diagnosis. Panel B: Dominant vs. non-dominant handgrip asymmetry by PTSD diagnosis.

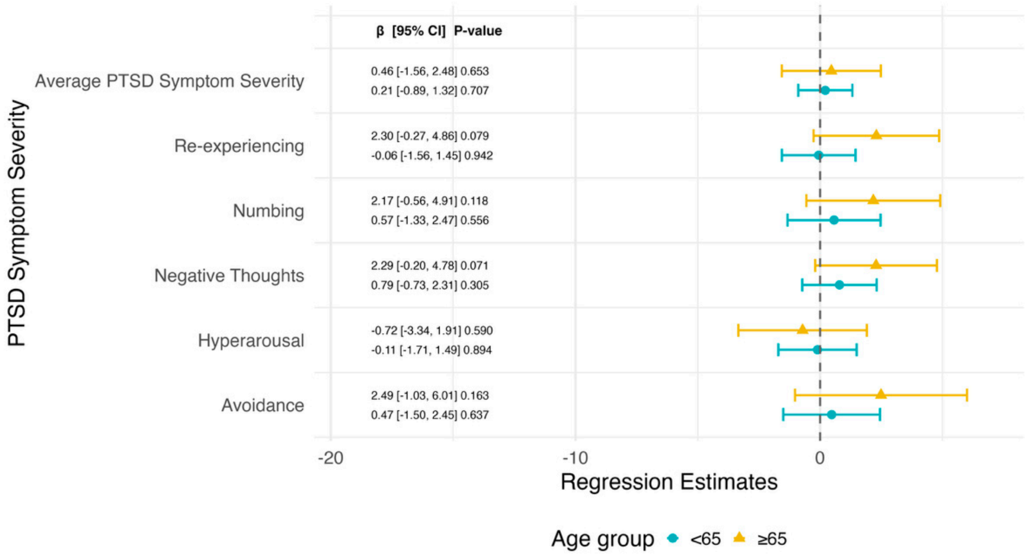

Figure S4. Multivariable-adjusted linear regression estimates showing the associations between PTSD symptom severity and absolute left-to-right handgrip asymmetry in 381 male responders from the Fire Department of the City of New York (FDNY), stratified by age group (<65 vs. ≥65). Estimates are shown by age group: <65 (bottom circle) and ≥65 (top triangle).
